# The first *de novo* genome assembly and sex marker identification of Pluang Chomphu fish (*Tor tambra*) from Southern Thailand

**DOI:** 10.1016/j.csbj.2022.03.021

**Published:** 2022-03-23

**Authors:** Komwit Surachat, Panchalika Deachamag, Monwadee Wonglapsuwan

**Affiliations:** a Division of Computational Science, Faculty of Science, Prince of Songkla University, Hat Yai, Songkhla 90110, Thailand; b Molecular Evolution and Computational Biology Research Unit, Faculty of Science, Prince of Songkla University, Hat Yai, Songkhla 90110, Thailand; c Division of Biological Science, Faculty of Science, Prince of Songkla University, Hat Yai, Songkhla 90110, Thailand; d Center for Genomics and Bioinformatics Research, Faculty of Science, Prince of Songkla University, Hat Yai, Songkhla 90110, Thailand

**Keywords:** *Tor tambra*, *Tor douronensis*, Genome survey, Genetic marker, Whole-genome sequencing, *De novo* assembly

## Abstract

The *Tor* genus belongs to the group of cyprinid fish commonly known as mahseer. Although *Tor* species are rapidly declining in the wild, and some face extinction, ambiguities in species identification hinder their collection and conservation. We conducted a genome survey of male and female *Tor tambra* collected in Thailand. The genome sizes of the male and female fish were approximately 1,671 and 1,645 Mb, respectively, with repeat contents of approximately 33%. The heterozygosity ratios of the male and female fish, which were 0.34% and 0.39%, respectively, suggested that the sex of *T. tambra* is determined by the ZW system. A sex marker was identified *in silico* and confirmed by PCR amplification. The result indicated that *T. tambra* has a ZZ/ZW sex determination system. Subsequently, comparative genomic and phylogenetic analyses of *T. tambra* and other fish in the Cyprinidae family were performed to explore the genetic diversity and evolution of the species. We also assembled the complete mitochondrial genome sequences of the *T. tambra* collected in Thailand. A phylogenetic tree of different *Tor* species, constructed based on mitochondrial genome sequences, indicated that *T. tambra* was closely related to *T. tambroides.* We believe this is the first genome survey of a species from the *Tor* genus or Mahseer group. Our results may help identify *Tor* species, providing a reference for genetic studies of the *Tor* genus and other mahseer fish.

## Graphical abstract

**Figure:**
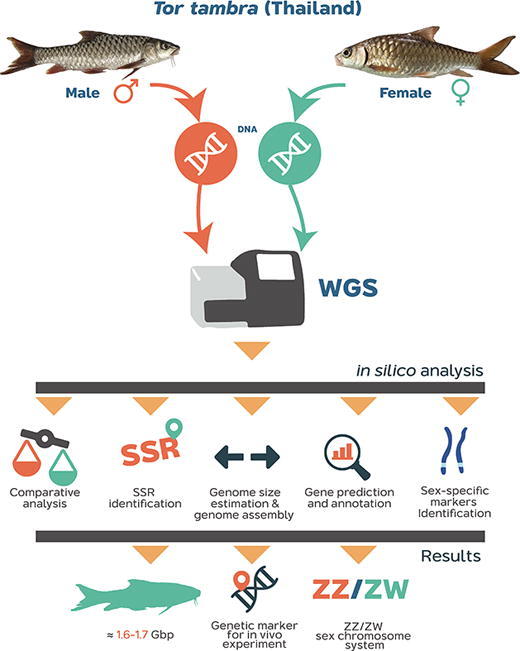

## 1 Introduction

Mahseer is the common name of a group of highly valuable freshwater fish in Asia. Mahseer fish have been classified into three genera: *Tor*, *Neolissochilus*, and *Naziritor*, but species of the *Tor* genus are commonly known as “true mahseers.” *Tor* species are generally attractive for sport fishing, have high commercial value as food, and bear religious and cultural significance in South and Southeast Asia. Despite the importance of these fish, their number in nature has been declining because of the destruction of their habitats and the construction of hydropower dams. Additionally, the conservation of *Tor* has been hindered by taxonomic ambiguity. The taxonomic debate and revision related to *Tor* species have been ongoing for a decade. As a result, *Tor* species are now included in the IUCN Red List. Three species are considered “near threatened,” one “vulnerable”, three “endangered,” and one “critically endangered”. However, eight species remain “data deficient” [1]. The authors agree with Jaafar and colleagues [2] on the need to sequence and study the whole-genome and transcriptome of the *Tor* species to prevent their extinction.

Among *Tor* species, the taxonomical classification of *Tor douronensis* is the most complex. *T. douronensis* has been classified as a synonym for *Tor tambra* [3], [4], which is among the species listed as “data deficient” by the IUCN. Although differences in the taxonomy and phylogeny of the two species have been demonstrated, the morphological and genetic comparisons between *T. tambra* and *T. douronensis* and their validity need to be further elucidated [4], [5]. The confusion involves not only the scientific names but also common names. This uncertainty is significant since the phylogenetic tree does not include *T. tambra* and *T. douronensis* from Thailand [5], [6], where *T. douronensis* is known as “*pla pluang chomphu*,” which means “pink color fish.” This description is at odds with the literature, which records that *T. douronensis* has a distinctive silvery and yellowish body color, whereas *T. tambra* has a distinctive reddish body color [7]. Since the distinction between these two species is still debated and *T. douronensis* is still an invalid name, we have used the valid name *T. tambra* [8] in the present work, to refer to the fish known in Thailand as “*pla pluang chomphu*”, which we collected from Narathiwat Province in the south of the country.

Next-generation sequencing (NGS), a powerful tool for generating genomic information, has been used in the genomic and evolutionary studies of various fish species. Genome surveys help us understand genome evolution, providing important genetic data. Information about genome size, repeat ratio, GC content, heterozygosity ratio, microsatellite information, and mitochondrial genome organization helps us design various experiments during the whole-genome study of an organism. Moreover, NGS has been used to analyze the mitochondrial genome sequences in a few *Tor* species, such as *Tor khudree*, *Tor tor*, *Tor putitora*, and *Tor tambroides* [6], [7], [8], [9]. However, a complete genome survey of a species from the *Tor* genus has not yet been reported.

Therefore, in this study, we investigated the genome sequence of *T. tambra* found in Thailand through *de novo* genome assembly, annotation, comparative genome analysis, and identification of sex-specific markers. To the best of our knowledge, this is the first report of a genome survey of a member of the *Tor* genus. Our data may be useful for the genetic identification of *Tor* species and may provide a guideline for conducting other genome surveys of related species.

## 2 Materials and methods

### 2.1 Fish specimens

One male and one female *T. tambra* were obtained from the Chanae District, Narathiwat Province, Thailand. The fish were handled according to the guidelines of the Animal Ethics Committee of Prince of Songkla University, Hat Yai, Songkhla, Thailand.

### 2.2 Histology

Following anesthesia of *T. tambra* with 2-phenoxyethanol (Sigma-Aldrich), the gonads were removed and immediately dissected for histological analysis to confirm the sex of the fish. The gonads were then chopped into smaller pieces and fixed in Bouin’s fixative at 4 °C for 7 h. After fixation, tissues were washed and stored in 70% ethanol, followed by dehydration in an alcohol gradient and xylene. Dehydrated gonads were then paraffin-embedded and sliced into 5-µm-thick sections prior to hematoxylin and eosin staining.

### 2.3 DNA extraction

Genomic DNA was isolated from muscle tissues of each specimen using a QIAquick Genomic DNA kit (QIAGEN, USA) according to the manufacturer’s protocol. Briefly, 15 mg of muscle tissue was incubated at 55 °C overnight with 600 µL of cell lysis solution and 60 µg of proteinase K. Then, 12 µg of RNase A was added to the sample tube, followed by incubation at 37 °C for 30 min. Proteins were precipitated by adding 200 µL of protein precipitation solution, followed by incubation in ice for 15 min. Sample tubes were centrifuged at 13,200 rpm for 15 min at 4 °C, and the supernatant was transferred to a new tube. DNA was precipitated by the centrifugation of the supernatant with the addition of 600 µL of cold isopropanol. The DNA pellet was washed with 70% ethanol and centrifuged at 13,200 rpm for 10 min at 4 °C. The DNA was suspended in deionized water and stored at −20 °C until further use.

### 2.4 Library preparation and whole-genome sequencing

Two paired-end genomic libraries (one from the male specimen and one from the female) with an insert size of 350-bp were constructed using the TruSeq Nano DNA Kit (San Diego, California, USA) following the manufacturer’s instructions. DNA libraries were then sequenced using the Illumina NovaSeq 6000 sequencing system from Macrogen (Seoul, Korea) to obtain 2 × 150-bp paired-end reads.

### 2.5 Genome size estimation and de novo genome assembly

The raw data were filtered using the NGS QC Toolkit [10] to remove low-quality reads and adapter sequences. The k-mer frequency and genome coverage were determined using jellyfish script [11]. The resulting histogram was used as an input for GenomeScope [12] to calculate the genome size, repeat content, and heterozygosity rate. Genome assemblies were generated from the filtered reads by SOAPdenovo2 [13] with k-mer values ranging from 21 to 63. The k-mer value of 45 produced the assembly with the highest N50, so 45 was the k-mer value used in constructing the final *de novo* assembly of *T. tambra*, which was generated by SOAPdenovo2 with default parameters.

### 2.6 Assembly quality assessment and gene prediction

The quality assessment of the male and female genome assemblies was performed using QUAST [14], inputting different k-mer values. The quality of the resulting assemblies was then compared. The AUGUSTUS program [15] was applied using default parameters for gene prediction, and data regarding *Danio rerio* (zebrafish) were used as the training dataset. Functional annotation was performed using eggNOG-mapper [16], [17] with default parameters. The completeness of the genome assembly was determined by BUSCO [18], [19] using the cyprinodontiformes_odb10 database in the assessment.

### 2.7 Comparative genomic analysis

To perform a comparative genomic analysis of related species, we first randomly selected ten scaffolds (length > 5000 bp) from both male and female draft assemblies and searched against the NCBI Reference Sequence Database (refseq_genomes), setting the target organism as fish (taxid: 7898). We selected the first top-six hits with the highest similarity and coverage and downloaded those assemblies (Table S1) for further comparative analysis. All downloaded genomes were indexed, and our draft assemblies were aligned against the indexed genomes using the Burrows-Wheeler Alignment (BWA) tool [20], [21]. All statistics were then retrieved for comparison using SAMtools [22]. The single-copy orthologous genes shared by all fishes were extracted from BUSCO analysis results to construct a phylogenetic tree using a Python script (BUSCO_phylogenomics.py) [23], [24] with 1000 bootstrap replicates and *D. rerio* as an outgroup.

### 2.8 Identification of simple sequence repeats (SSRs)

The SSRs in the *de novo* genome assemblies were identified using the MISA tool [25]. Mono-, di-, tri-, tetra-, penta-, and *hexa*-nucleotide microsatellites with a minimum of 12, 7, 5, 5, 5, and 5 repetitions, respectively, were identified [26], [27].

### 2.9 Mitochondrial genome assembly and phylogenetic analysis

**Fig. 1:**
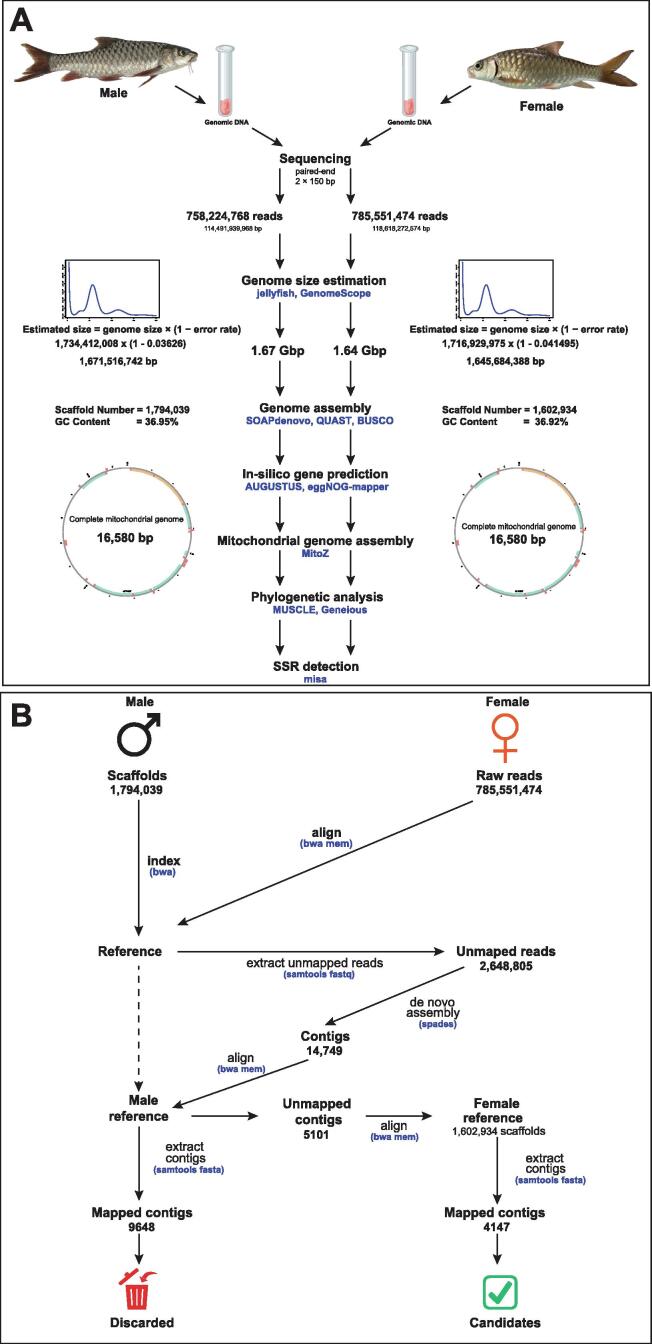
An overview of the experimental design and bioinformatics analysis pipeline. (A) Bioinformatics workflow for genome size estimation, assembly, and annotation. (B) *In silico* identification of sex-specific markers based on an NGS approach to data analysis.

To assemble and analyze mitochondrial genome sequences, we used MitoZ [28], a Python3-based toolkit that automatically assembles, finds mitogenome sequences, annotates, and provides visualizations in a single command. Furthermore, complete mitochondrial DNA sequences (mtDNA) of 24 *Tor* species and one *Neolissochilus* species (Table S2) were downloaded from the NCBI database, and all gene and rRNA sequences were extracted from each mtDNA sequence. The mtDNA sequences and all individual gene sequences (*ATP6*, *ATP8*, *COX1-3*, *NAD1-6*, *ND4L,* 12S rRNA, and 16S rRNA) from mitogenomes were independently subjected to multiple sequence alignment (MSA) using MUSCLE [29]. The MSA results were used for phylogenetic tree construction using the neighbor-joining method with a bootstrap value of 1,000 in Geneious software [30]. In addition, to visualize the genomic structure among mitogenomes, a comparative map was then constructed using the MAUVE software [31]. An overview of the complete bioinformatics analysis workflow is shown in Fig. 1A.

### 2.10 In silico identification of candidate W chromosome-specific DNA fragments

To obtain candidate, specific DNA sequences on the W chromosome of *T. tambra* for use as a sex marker, we applied an NGS-based approach as previously proposed by Ou et al. [32]. Briefly, we first mapped DNA sequences against the reference genome by aligning raw sequence reads from female fish to the male draft assembly with the BWA tool [20], [21]. Next, unmapped reads were extracted from the alignment file (.bam) using SAMtools [22], and *de novo* assembly was performed using SPAdes [33]. Contigs shorter than 200 bp were discarded. The retained contigs from the *de novo* assembly were then realigned to the male draft assembly to identify sequences matching male DNA fragments and exclude them from further analysis. Since only unaligned sequences could align to the female reference, retrieved mapped sequences constituted the set of possible female-specific fragments on the W chromosome. The overall analysis workflow is illustrated in Fig. 1B.

### 2.11 PCR amplification for confirmation of sex marker DNA fragments

We randomly selected 50 DNA sequences from a set of female-specific fragments retrieved in previous analyses. Using these sequences, primers were designed *in silico*, using Primer3 [34]. The designed primers were then tested by individually aligning primer sequences to male and female reference assemblies using BWA [20], [21]. Primer sequences that mapped to the male reference, did not map to the female reference or only mapped to the female on one side of the primer pair were discarded. Thirteen pairs of primers were selected to be used for PCR amplification.

Three individual males and three individual females were used as templates. Genomic DNAs were extracted from the fin according to the DNA extraction protocol (please refer to section 2.3). The PCR reaction consisted of 500 ng gDNA, 250 mM dNTPs, 1 pmol of each primer, 2.5 mM MgCl_2_, 1x GoTaq® Flexi buffer, and 0.25 units GoTaq Flexi DNA polymerase (Promega, USA). Initial cycling was at 95 °C for 3 min, followed by 30 cycles of each primer’s annealing temperature and annealing time, with a final elongation step at 72 °C for 5 min. The primer sequences, annealing temperatures, and annealing times are listed in Table S3.

## 3 Results and discussion

### 3.1 Morphology and histology of T. Tambra specimens

**Fig. 2:**
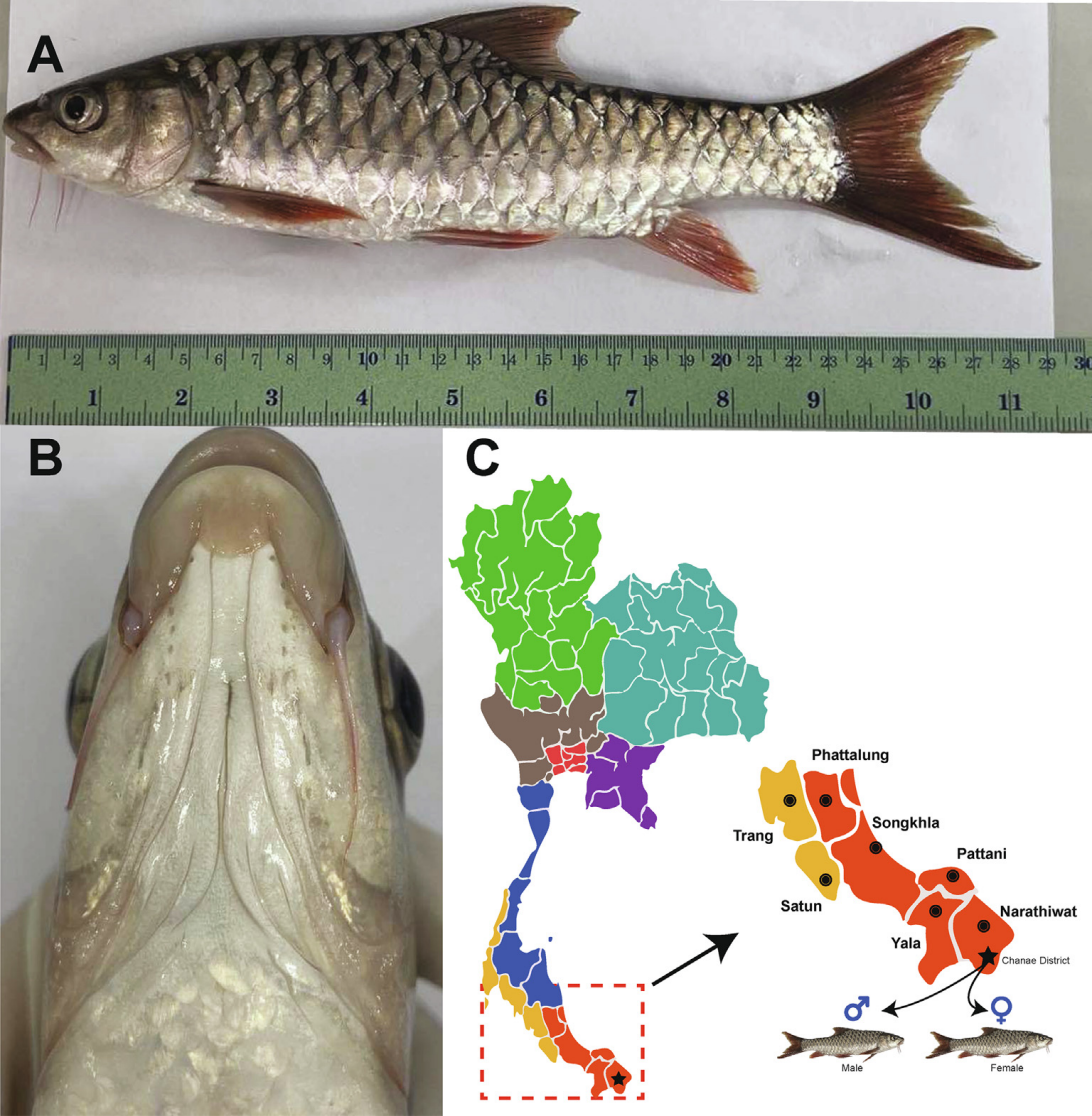
Morphological features of *T. tambra* and its sampling site location. (A) *T. tambra* specimen collected from Narathiwat Province, Thailand. (B) Median lobe of *T. tambra*. (C) Sampling site location.

**Fig. 3:**
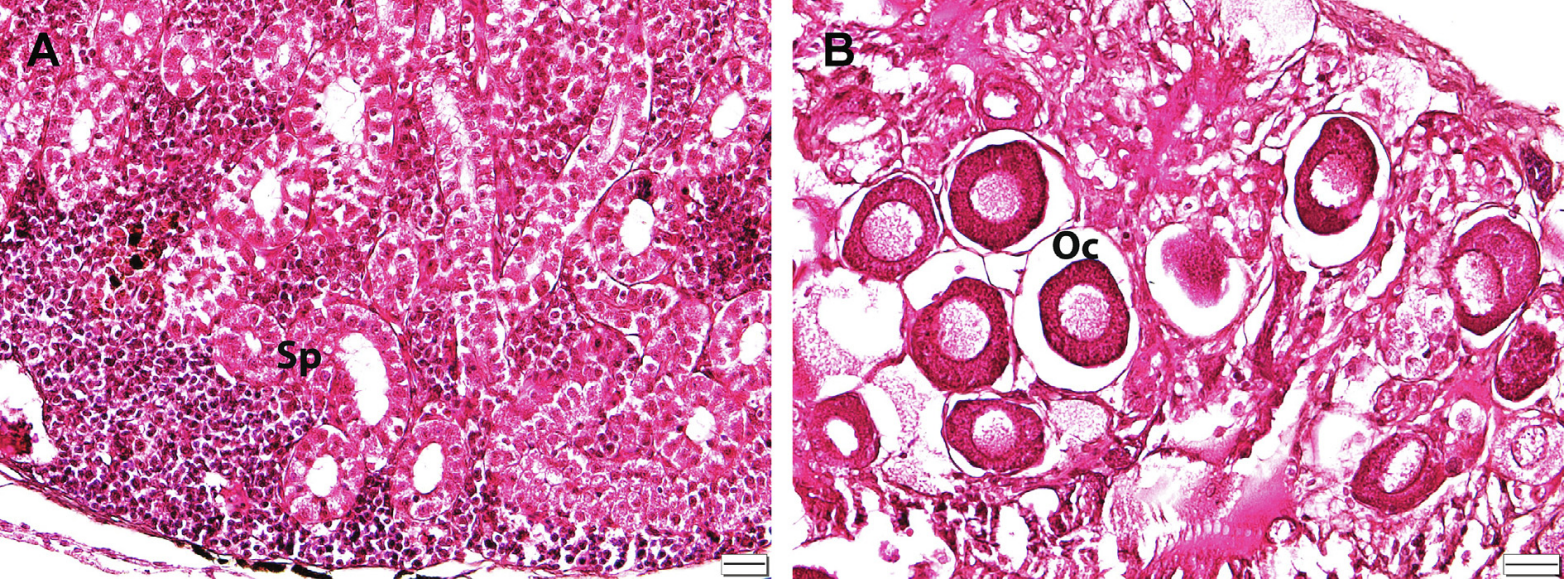
Sex determination of the specimens using histological staining of the gonads. (A) Stained tissue section of male *T. tambra* testis. (B) Stained tissue section of female *T. tambra* ovary. Sp; spermatogonia, Oc; oocyte. Scale bar = 20 µm.

Photographs of a specimen collected from Narathiwat Province, Thailand, are shown in Fig. 2A and 2C. The fish had 23 lateral scales and a lower median lobe (Fig. 2B), a distinctive characteristic of *Tor* species. The mouth was sub-terminal, which is consistent with the morphology of *T. tambra* [9]. Typically, the male *T. tambra*, but not the female, has tubercles covering virtually the entire side of the face, with the number of tubercles depending on the fish size. The tubercles in small fish are almost invisible [3]. Therefore, the sex identification of small males can be uncertain. For accuracy, we determined the sex of the specimens based on histological analysis of the gonads. Fig. 3 shows the stained histological sections of the ovaries and testes of collected specimens showing immature oocytes and spermatogonia, respectively. For sequencing experiments, muscle tissues from the male and female fish were used.

### 3.2 Genome sequencing statistics

We obtained a total of 140.4 Gb and 134.5 Gb of raw sequencing data for the male and female specimens, respectively. The Phred quality scores from both libraries reached 20 (Q20) and 30 (Q30), approximately 96% and 91% of all raw reads, respectively. After the quality analysis, > 98% of the good quality reads from each library were retained (Table S4). The genome size of *T. tambra* is unknown because whole-genome sequences in the *Tor* genus are unavailable. Only mitochondrial genome surveys have been conducted in genomic diversity studies of *Tor* species across Southeast Asia, China, and India [9], [35], [36]. Comparative genome analysis of 52 fish species [37] revealed that genomes of the species in the order Cypriniformes ranged in size from 1.2 to 1.8 Gbp. We, therefore, hypothesized that the *T. tambra* genome should be less than 2.0 Gbp. In the present study, we considered a data depth of approximately 120–140 Gb and obtained a sequencing depth of approximately 84x and 82x for the male and female fish, respectively.

### 3.3 Genome size estimation

**Fig. 4:**
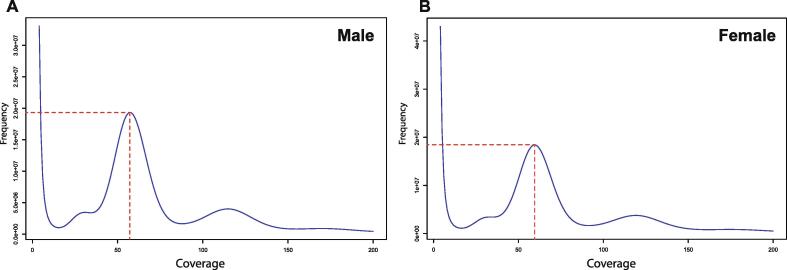
Coverage and distribution frequency of 17-mer in (A) male and (B) female *T. tambra* reads. The x-axis represents the coverage, whereas the y-axis represents the distribution frequency.

To estimate the genome size, a k-mer analysis was performed using the filtered raw reads. We used a 17-mer frequency distribution to plot k-mer coverage. The analysis resulted in k-mer distribution curves with peaks at 57x for the male specimen (Fig. 4A) and 60x for the female (Fig. 4B). The genome size of male and female *T. tambra* was calculated using the following formula: Genome size = K-mer num / K-mer depth [11], yielding respective estimations of 1.734 Gb and 1.716 Gb. Then, revised genome size was calculated using the following approach to exclude the k-mer errors: Revised genome size = genome size × (1 − error rate). Table 1 shows the overall statistics of genome size estimation. The revised estimated genome size of the male specimen was slightly larger (by 25.84 Mb) than that of the female, while the heterozygous ratio and repeat content of the male fish were slightly smaller than those of the female. Thus, our results could imply a previously unreported variation in the sex chromosome system of *Tor* species. Furthermore, since the heterozygosity ratio was higher and the genome size smaller in female fish, we suggest that the sex system of *T. tambra* should be a ZW system. Feller and colleagues discussed the relationship between sex chromosome systems, genome size, and the heterozygosity ratio, indicating that if the sex chromosomes of *T. tambra* were heteromorphic, the genome size of male and female fish would differ due to the degeneration of Y or W chromosomes. Moreover, a higher heterozygous ratio in males indicates an XY system, whereas a higher heterozygous ratio in females indicates a ZW system [38]. Thus, based on the data presented here, the sex chromosome of *T. tambra* males should be ZZ owing to the larger genome size of the male, while for females, it should be ZW owing to the higher heterozygous ratio of the female.

**Table 1:** Genome size estimation based on K-mer statistics.

Fish specimen	K-mer depth	Genome size (Mb)	Revised genome size (Mb)	Heterozygous ratio (%)	Repeat (%)
Male	57	1734.41 (1,734,412,008 bp)	1671.52 (1,671,516,742 bp)	0.34	32.6
Female	60	1716.93 (1,716,929,975 bp)	1645.68 (1,645,684,388 bp)	0.39	32.8

The genomes of only a few cyprinid species have been sequenced. These include *Pimephales promelas* (1.22 Gb) [39], *Sinocyclocheilus rhinocerous* (1.66 Gb) [40], *Sinocyclocheilus graham* (1.75 Gb) [40], *Sinocyclocheilus anshuiensis* (1.63 Gb) [40], *Cyprinus carpio* (1.72 Gb) [41], and *D. rerio* (1.68 Gb) [42]. A literature review revealed no whole-genome surveys of *Tor* species or even a closely related species, and this is the first study to provide an accurate genome sequence for *T. tambra*.

### 3.4 De novo genome assembly and gene prediction

The genome assemblies of male and female *T. tambra* were constructed independently using k-mer values ranging from 21 to 63. The best assembly was selected based on the N50 value. We obtained male and female genome assemblies containing a total of 1,794,039 and 1,602,934 scaffolds with an N50 value of 1458 and 2057, respectively (Supplementary Table S5). The average GC contents of the male and female *T. tambra* assemblies were 36.95% and 36.92%, respectively. Furthermore, male and female fish assemblies contained approximately 33% repeats (Table 1). It should be noted that the repeat contents directly affected the assembly process. Since they could not be anchored in the scaffolds, a large number of scaffolds with low N50 values were produced, likely underestimating repeat rates and highlighting the complexity of the *T. tambra* genome*,* which cannot be completed only with short reads.

Based on predictions from the AUGUSTUS software, a total of 91,407 male and 104,938 female genes were predicted from the assembled genomes with 167,692 and 222,203 introns, respectively. The number of predicted genes of both sexes of *T. tambra* was higher than that of annotated genes of related species, such as *C. carpio* (≈ 53,000) [41] and *Carassius auratus* (75,736) [43]. However, the number of predicted genes of *T. tambra* could be unusually high because the genomes broke into many contigs, affecting the performance of the gene identification algorithm in the prediction process. This could directly influence the number of predicted genes compared to other organisms. In addition, the genome completeness of *T. tambra*, evaluated by BUSCO, was less than 12%, while for other related organisms, completeness ranged within 60–67% (Fig. S1). High-quality genome and transcriptome sequencing should be conducted in the future to improve the *T. tambra* draft genome assembly and annotation.

**Fig. 5:**
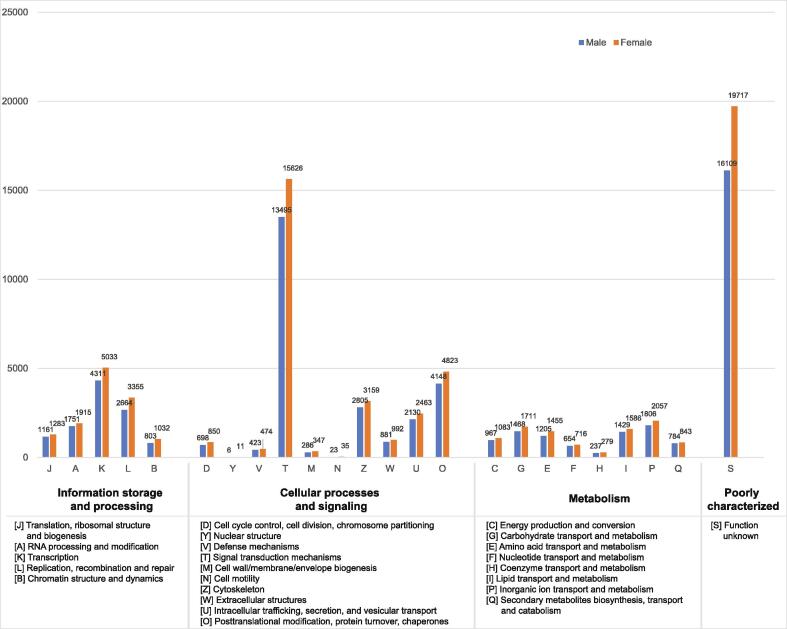
Functional annotation of male and female *T. tambra* based on KOG categorization. The x-axis indicates the category of KOG classification. The y-axis indicates the number of genes in each category.

Functional annotation was then performed using several databases, including KOG, Gene Ontology (GO), Kyoto Encyclopedia of Genes and Genomes (KEGG), and PFAM (Table S6). Using the selected databases, we could assign over 50% of functional annotations from both *T. tambra* samples. In the annotation from the KOG database, unsurprisingly, the most abundant category was poorly characterized annotations (25%). The cellular processes and signaling categories were found with the second-highest frequency in both sexes. Over 50% of genes classified in the signaling category related to signal transduction mechanisms (Fig. 5).

This first genome survey of a *Tor* species provides genomic information and a useful guideline for future complete genome analyses of related species. However, owing to the large size of the *T. tambra* genome and the high repeat content, it may be prohibitive to sequence mate-pair and long-read libraries to obtain better repeat annotation and Hi-C data to build the 3D architecture of the genome [44].

### 3.5 Comparative genomic and phylogenetic analyses

We first investigated the similarity of assembly results between *T. tambra* and other organisms. We randomly selected scaffolds longer than 5000 bp from both male and female assemblies for a megablast search against the NCBI database. The identification results are summarized in Table S7. The highest identity and coverage returned from the search were from the Cyprinidae, *S. anshuiensis*, *S. rhinocerous*, *S. grahami* (golden-line barbel), *C. auratus* (goldfish), *Puntigrus tetrazona* (Sumatra barb), and *C. carpio* (common carp). The percent identities of the results from those six organisms ranged from 80 to 97; however, the coverages from the identifications varied from 10% to 87%. We then selected these six genomes and one model organism (*D. rerio*) to perform an alignment analysis to identify regions of similarity between *T. tambra* and other species. Our draft assemblies of male and female *T. tambra* were aligned against seven reference organisms. The highest coverage percentage from the alignment came from *P. tetrazona* (≈43%), while the lowest coverage was from *D. rerio* (≈9%) (Table S8). In contrast, over 60% of all sequences could not be aligned into these genomes, implying genetic variations between *T. tambra* and other fishes in the same family.

**Fig. 6:**
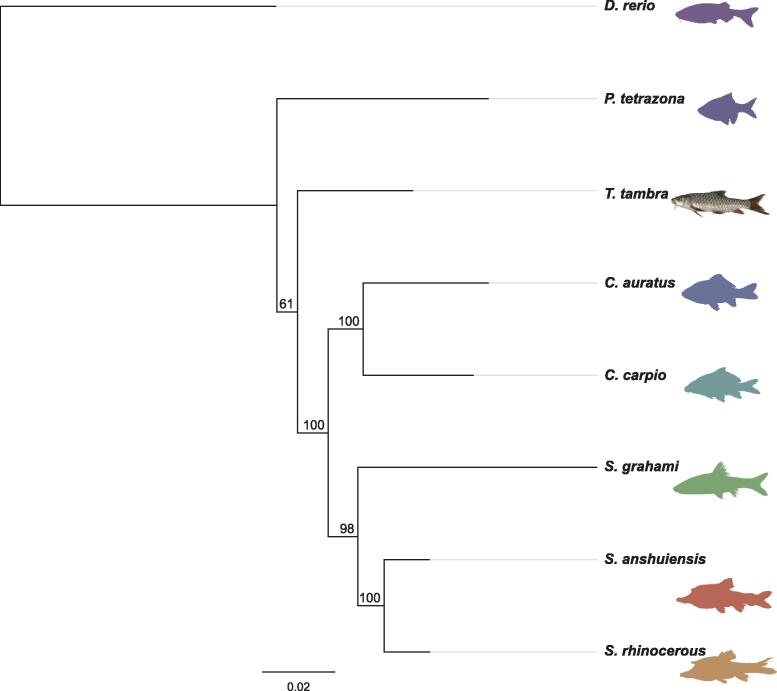
Phylogenetic tree based on 16 single-copy orthologous genes extracted from BUSCO analysis of *T. tambra* and other seven fishes in the family Cyprinidae.

To explore the phylogenetic relationship between *T. tambra* and reference species, we constructed a phylogenetic tree based on a set of single-copy genes retrieved from the BUSCO pipeline. Sixteen retrieved single-copy orthologous protein sequences common to all the species were used (Fig. 6). The zebrafish formed a cluster distinct from the other seven species. The three *Sinocyclocheilus* species occupied the same clade as *C. carpio* and *C. auratus* while *T. tambra* and *P. tetrazona* clustered separately. Although all these organisms in the tree belong to the same family, the phylogenetic relationships and genetic distances evidenced explicit differences. Although most of the organisms used in this analysis were well annotated and recently sequenced in high-resolution, genome sequences from the *Tor* genus are not so well researched. Only DNA fragments and mtDNA have been sequenced. Nonetheless, this genome survey provides new insight into the evolutionary and phylogenetic relationships between *T. tambra* and other species in the same family.

### 3.6 Microsatellite motif identification

Microsatellites or SSRs are repetitive DNA sequences of 1–6 nt motifs found in eukaryotes [19]. SSR markers have been used as molecular markers for animal identification [9] and plant breeding [20]. We found 1,818,192 and 1,614,239 SSRs in the male and female *T. tambra* genomes, respectively (Table 2). Among these SSRs, the mononucleotide SSR motifs were the most abundant in both male and female fish, accounting for 43.01% and 40.56%, respectively (Supplementary Fig. S2). Microsatellite markers reportedly help identify *Tor* species but could not resolve the ambiguity between *T. douronensis* and *T. tambroides* [45], [46]. Therefore, the identification of a higher number of SSRs in more *Tor* species may help improve species identification and our understanding of the evolution of the genus.

**Table 2:** Microsatellite motifs identified in *T. tambra.*

SSR summary	Female	Male
Total number of sequences examined	3,837,459	4,362,067
Total size of examined sequences (bp)	1,956,963,080	1,871,747,511
Total number of identified SSRs	1,614,239	1,818,192
Number of SSR containing sequences	1,026,805	1,284,940
Number of sequences containing more than one SSR	321,212	330,918
Number of SSRs present in compound formation	317,264	335,762

### 3.7 Mitochondrial genome assembly

**Fig. 7:**
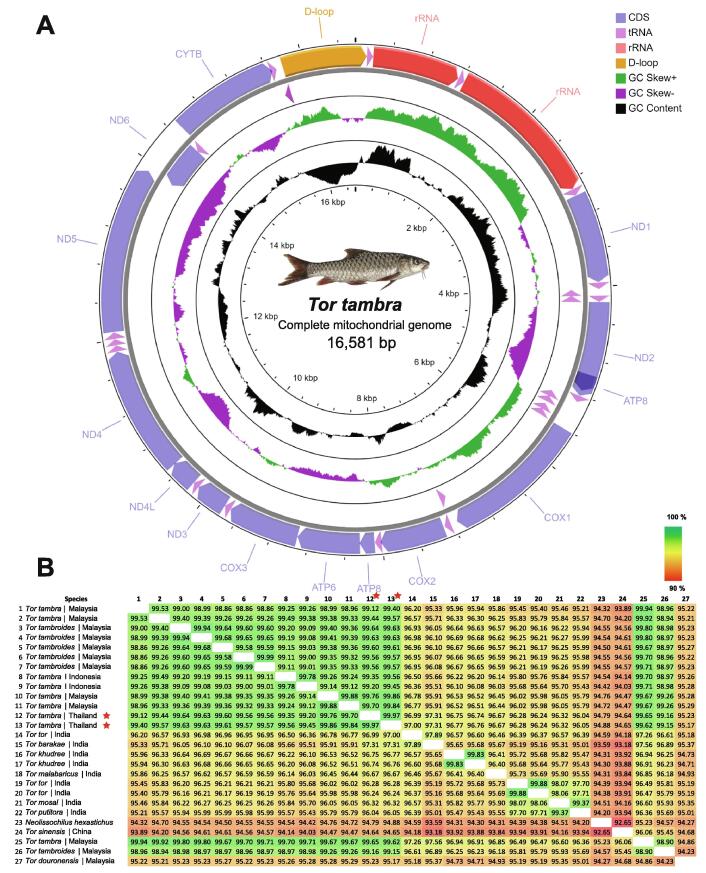
Mitochondrial genome of *T. tambra*. (A) Mitochondrial genome organization (B) Average nucleotide identity (ANI) of mitochondrial genome sequences from different *Tor* species and *Neolissochilus hexastichus*.

**Fig. 8:**
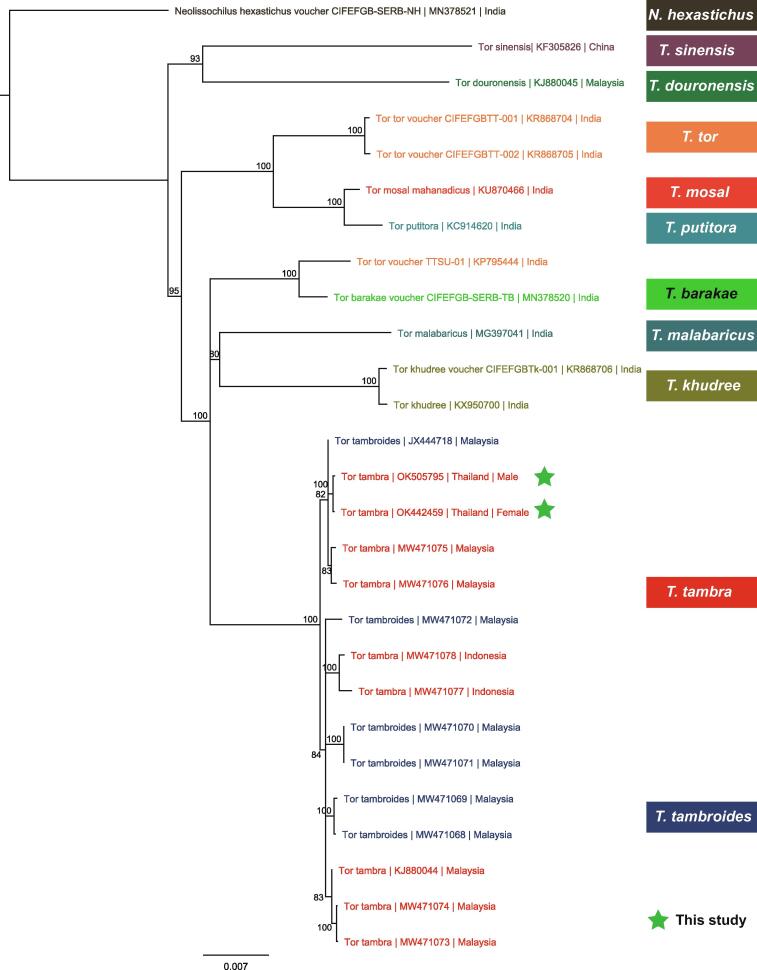
Phylogenetic tree representing different *Tor* species and *N. hexastichus* constructed using whole mtDNA sequences with 1000 bootstrap replicates.

In both male and female *T. tambra*, the typically circular mtDNA was approximately 16.5 kb in length and encoded 13 coding sequences, 22 tRNAs, two rRNAs, and one D-loop (Fig. 7A). To perform a comparative analysis and construct a phylogenetic tree, complete mitochondrial genomes of all *Tor* species and *N. hexastichus* were retrieved from the NCBI database. The retrieved mtDNA sequences ranged between 16,554 and 16,780 bp. The GC content was found to be approximately 43% across all species. The pairwise average nucleotide identity (ANI) of all mtDNAs was > 92% (Fig. 7B), and the male and female *T. tambra* considered in the present study shared the highest similarity with *T. tambra* and *T. tambroides* (99.7% and 99.2%, respectively) from Malaysia. Additionally, MSA of the mtDNAs was performed to further analyze the mitogenome synteny and arrangement among *Tor* species (Fig. S3). A phylogenetic tree was constructed based on whole mtDNA sequences of 27 species. The male and female *T. tambra* clustered with *T. tambra* and *T. tambroides* from Malaysia (Fig. 8). We noted that the *Cytb* gene of *T. tambroides* from the mtDNA sequence (JX444718) was shorter (1,011 bp) than that of other species, approximately 1,141 bp (Fig. S4 and S5). Of note, the short *Cytb* gene might have affected the clustering of *T. tambroides* with *T. tambra.* Therefore, we constructed another phylogenetic tree using only the *Cytb* gene sequences from *T. tambroides* and other *Tor* species. Although *T. tambra* and *T. tambroides* clustered together, the *T. tambroides* (JX444718) showed the longest distance in the cluster. In addition, we constructed phylogenetic trees of other individual genes to explore genetic distances among different genes. Most of them separated *T. tambra* and *T. tambroides* from the other *Tor* species, but the whole mtDNA tree revealed much more detail about genetic distance than the single-gene trees (Fig. S6)*.* Therefore, we suggest that either complete mitogenomes or a high number of genetic markers combined with morphological taxonomy should be used to improve the classification of *Tor* species and resolve the ambiguities within this genus. However, a nuclear DNA marker might be needed to distinguish *T. tambra* and *T. tambroides* since they are very closely related and could not be differentiated with any marker from the mtDNA sequences.

### 3.8 Identification and confirmation of candidate W chromosome-specific DNA fragments

**Fig. 9:**
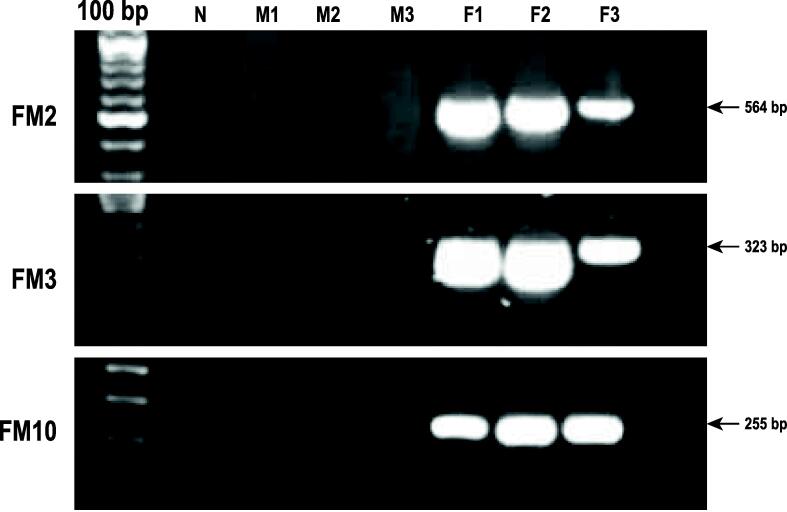
Validation of five candidate female-specific markers. Label M represents male *T. tambra.* F represents female *T. tambra*. The PCR products were visualized by 1.5% gel agarose electrophoresis.

Since genome size estimation revealed that the male genome was larger than the female genome while the heterozygous ratio was lower in the female genome, we hypothesized that *T. tambra* should have a ZW sex determination system. We then identified a set of candidate W chromosome-specific DNA fragments based on this hypothesis. We mapped the female sequence reads to male scaffolds and extracted only unmapped reads for further use. All unmapped reads were reassembled, resulting in 14,749 contigs with a maximum length of 7636 bp. The distribution of contig length from the assembly is shown in Fig. S7. All contigs were remapped to the male reference to exclude non-specific DNA fragments for use as female markers. As a result, only 5101 contigs survived the alignment, of which 4147 contigs could be mapped back to the female reference. From the remainder, we randomly selected 50 contigs to design primers that were tested against the male and female references following the aligning method described above. Thirteen pairs of designed primers mapped only to the female reference, while others mapped either to both sexes or the female reference only on one side of the primer pair. Therefore, we confirmed the specificity of those 13 pairs of primers with PCR amplification. The results are given in Fig. 9. The FM2, FM3, and FM10 primer pairs were female-specific. The FM2, FM3, and FM10 primers produced a 564, 323, and 255 bp band in all females, respectively. Nine of thirteen primers showed PCR products in both male and female DNA, and one pair of primers displayed no band in any of the samples. This might cause by the assembly of male and female references since the assembled results of both libraries produced many scaffolds and yielded very low N50. Some DNA fragments might be discarded in the male reference resulting in false positive in the *in silico* testing. In contrast, one primer did not yield any band in male and female fishes. This could be some errors in generating contigs since several candidate female contigs could be aligned back by raw female reads with coverage of approximately 90%. The female-specificity of the FM2, FM3, and FM10 primers revealed female fish-specific DNA regions. We believe that these specific regions might be in the W chromosome. Therefore, the higher female heterozygote ratio and the existence of a female-specific DNA region support our hypothesis that the sex of *T. tambra* is determined by a ZW system. However, a karyotypic investigation should be performed in the future to confirm the sex system of *Tor* species.

## 4 Conclusions

We assembled and annotated the complete genome sequence of the freshwater fish species *Tor tambra*. To the best of our knowledge, this is the first genome survey of any species in the *Tor* genus. We assembled the whole-genome sequences of male and female fish specimens. The male and female genome sizes were 1.67 GB and 1.64 GB, respectively, with approximately 33% repeat sequences in both genomes. A sex marker was also identified using an NGS-based approach. Differences between male and female fish in genome size and heterozygosity ratio, as well as our *in silico* sex marker identification, imply that the sex chromosome system of *T. tambra* is ZZ/ZW. Comparative genome and phylogenetic analyses revealed genetic diversity and variation among fishes in the Cyprinidae family. Over 60% of nucleotide sequences of *T. tambra* could not be aligned to any other closely related genomes. This finding could lead to discovering new genetic elements after sequencing at a better resolution. Based on comparative mitochondrial genome analysis of *T. tambra* against other *Tor* species, we concluded that *T. tambra* was closely related to *T. tambroides* and that the two species could not be distinguished using mtDNA or any mitochondrial marker. We suggest that the identification of these two species should be clarified using nuclear DNA sequences as genetic markers. The results of the current study provide important insights for further genetic analyses of species in the *Tor* genus and mahseer fish generally.

## CRediT authorship contribution statement

**Komwit Surachat:** Conceptualization, Data curation, Formal analysis, Investigation, Methodology, Resources, Software, Validation, Visualization, Writing – original draft, Writing – review & editing. **Panchalika Deachamag:** Investigation, Methodology, Validation. **Monwadee Wonglapsuwan:** Conceptualization, Data curation, Formal analysis, Investigation, Methodology, Resources, Project administration, Supervision, Funding acquisition, Validation, Visualization, Writing – original draft, Writing – review & editing.

## Declaration of Competing Interest

The authors declare that they have no known competing financial interests or personal relationships that could have appeared to influence the work reported in this paper.
