# Supplementary material for: The first de novo genome assembly and sex marker identification of Pluang Chomphu fish (Tor tambra) from Southern Thailand
Source: Comput Struct Biotechnol J. 2022 Mar 23;20:1470–80. doi: 10.1016/j.csbj.2022.03.021 (PMC8976102; doi:10.1016/j.csbj.2022.03.021)
Supplement: Supplementary data 1 [file mmc1.docx]

**Supplementary materials**

**Table S1 Fish genome sequences used in the comparative analysis**

| **No.** | **Common name** | **Scientific name** | **Sequence Length** | **Assembly level** | **Accession** |
| --- | --- | --- | --- | --- | --- |
| 1 | Common carp | *Cyprinus carpio* | 1,713,658,011 | Chromosome | GCF_018340385 |
| 2 | Goldfish | *Carassius auratus* | 1,820,635,050 | Chromosome | GCF_003368295 |
| 3 | Bony fishes | *Sinocyclocheilus anshuiensis* | 1,632,718,266 | Scaffold | GCF_001515605 |
| 4 | Bony fishes | *Sinocyclocheilus rhinocerous* | 1,655,786,410 | Scaffold | GCF_001515625 |
| 5 | Golden-line barbel | *Sinocyclocheilus* *grahami* | 1,750,287,761 | Scaffold | GCF_001515645 |
| 6 | Sumatra barb | *Puntigrus tetrazona* | 730,818,536 | Chromosome | GCF_018831695 |
| 7 | Zebrafish | *Danio rerio* | 1,679,203,469 | Chromosome | GCF_000002035 |

**Table S2 Mitogenomes used in this study**

| **No.** | **Organism** | **Sequence Length** | **%GC** | **Country** | **Accession** |
| --- | --- | --- | --- | --- | --- |
| 1 | *Tor tor* | 16,571 | 43.00 | India | KR868704 |
| 2 | *Tor tor* | 16,573 | 43.00 | India | KR868705 |
| 3 | *Tor tor* | 16,554 | 43.10 | India | KP795444 |
| 4 | *Tor tambroides* | 16,690 | 42.80 | Malaysia | JX444718 |
| 5 | *Tor tambroides* | 16,578 | 42.90 | Malaysia | MW471068 |
| 6 | *Tor tambroides* | 16,577 | 42.90 | Malaysia | MW471069 |
| 7 | *Tor tambroides* | 16,581 | 42.90 | Malaysia | MW471070 |
| 8 | *Tor tambroides* | 16,581 | 42.90 | Malaysia | MW471071 |
| 9 | *Tor tambroides* | 16,582 | 42.90 | Malaysia | MW471072 |
| 10 | *Tor tambra* | 16,581 | 42.90 | Malaysia | KJ880044 |
| 11 | *Tor tambra* | 16,518 | 43.00 | Malaysia | MW471073 |
| 12 | *Tor tambra* | 16,450 | 43.00 | Malaysia | MW471074 |
| 13 | *Tor tambra* | 16,553 | 43.00 | Malaysia | MW471075 |
| 14 | *Tor tambra* | 16,553 | 42.90 | Malaysia | MW471076 |
| 15 | *Tor tambra* | 16,507 | 43.00 | Indonesia | MW471077 |
| 16 | *Tor tambra* | 16,506 | 43.00 | Indonesia | MW471078 |
| 17 | *Tor tambra** | 16,581 | 42.90 | Thailand | OK505795 |
| 18 | *Tor tambra** | 16,581 | 42.90 | Thailand | OK442459 |
| 19 | *Tor sinensis* | 16,506 | 43.20 | China | KF305826 |
| 20 | *Tor putitora* | 16,576 | 43.10 | India | KC914620 |
| 21 | *Tor mosal mahanadicus* | 16,572 | 43.10 | India | KU870466 |
| 22 | *Tor malabaricus* | 16,580 | 43.30 | India | MG397041 |
| 23 | *Tor khudree* | 16,576 | 43.30 | India | KX950700 |
| 24 | *Tor khudree* | 16,573 | 43.30 | India | KR868706 |
| 25 | *Tor douronensis* | 16,586 | 43.40 | Malaysia | KJ880045 |
| 26 | *Tor barakae* | 16,780 | 42.90 | India | MN378520 |
| 27 | *Neolissochilus hexastichus* | 16,538 | 43.40 | India | MN378521 |

* This study

**Table S3** **Primers for sex identification**

| **Region** | **Primer name** | **Primer sequence** | **Annealing temp. and time** |
| --- | --- | --- | --- |
| FM1 | FM1-F | 5’- TTGGGACATGGCCATCAGAC - 3’ | 56 °c, 45 sec |
|  | FM1-R | 5’- GACCAGCCAAGAGAACAGCT - 3’ |  |
| FM2 | FM2-F | 5’- ATGCCTGCATTTACGGGTCA -3’ | 60 °c, 30 sec |
|  | FM2-R | 5’- GGACCGGCTCATCAGAGATG -3’ |  |
| FM3 | FM3-F | 5’- GCCAAACTCTTCCCAGGACA -3’ | 60 °c, 30 sec |
|  | FM3-R | 5’- TAGCACGTGCACATCACCAT -3’ |  |
| FM4 | FM4-F | 5’- CATGGATGAGGACCCTGCAT -3’ | 59 °c, 30 sec |
|  | FM4-R | 5’- CCCCCATGGTGTGAGTGTAG -3’ |  |
| FM5 | FM5-F | 5’- GCATTTGCAGCACTGTCTCC -3’ | 62 °c, 45 sec |
|  | FM5-R | 5’- AACGCTCACAGCATACGTGA -3’ |  |
| FM6 | FM6-F | 5’- GTACGAACGGACCAACCAGT -3’ | 64 °c, 30 sec |
|  | FM6-R | 5’- GCTTTGTCTTGCGCCATCAT -3’ |  |
| FM7 | FM7-F | 5’- CTGATGGAATTGGTGGGGCT -3’ | 62 °c, 30 sec |
|  | FM7-R | 5’- GCGGATACCTGGTCTGCTAC -3’ |  |
| FM8 | FM8-F | 5’- TCAAACTAGAGCGGCATCGT -3’ | 62 °c, 30 sec |
|  | FM8-R | 5’- TTTCCCCCTTTTCGACCTCC -3’ |  |
| FM9 | FM9-F | 5’- GGTCACACTGGTCAGACAGG -3’ | 62 °c, 30 sec |
|  | FM9-R | 5’- CGGGTAGCACAACACTCAGT -3’ |  |
| FM10 | FM10-F | 5’- AATGGTCAGCTTGGTGACGT -3’ | 63 °c, 30 sec |
|  | FM10-R | 5’- GAGCGCACGTCTTTGTGATC -3’ |  |
| FM11 | FM11-F | 5’- GGTATTCCTGGGGTTGTGGG -3’ | 60 °c, 30 sec |
|  | FM11-R | 5’- AGGGCATGCACTAAGTCCAC -3’ |  |
| FM12 | FM12-F | 5’- TGCCGGTTGACGCAGATAAA -3’ | 60 °c, 30 sec |
|  | FM12-R | 5’- CCTACCAGTGTTTCTGCCGA -3’ |  |
| FM13 | FM11-F | 5’- TTTTTAGCCGTGCCAAGTGC -3’ | 60 °c, 30 sec |
|  | FM11-R | 5’- TCGTGCGGAGGGATTTGAAA -3’ |  |

**Table S4 Genome sequencing statistics**

| **Fish specimen** | **Total reads** | **Raw read bases (bp)** | **Filtered read bases (bp)** | **GC (%)** | **Q20 (%)** | **Q30 (%)** |
| --- | --- | --- | --- | --- | --- | --- |
| Male | 785,551,474 | 118,618,272,574 | 116,493,463,457 | 38.25 | 96.37 | 91.19 |
| Female | 758,224,768 | 114,491,939,968 | 112,436,653,936 | 38.11 | 96.69 | 91.77 |

**Table S5 Statistics of *de-novo* assemblies of *T. tambra***

| **Fish specimen** | **Total length (bp)** | **Number of scaffolds** | **Max length (bp)** | **N50 (bp)** | **N75 (bp)** |
| --- | --- | --- | --- | --- | --- |
| Male | 1,532,111,938 | 1,794,039 | 35,899 | 1458 | 661 |
| Female | 1,640,426,230 | 1,602,934 | 38,479 | 2057 | 855 |

**Table S6 Functional annotation statistics**

| **Fish specimen** | **Male** | | **Female** | |
| --- | --- | --- | --- | --- |
|  | **Number** | **Percentage** | **Number** | **Percentage** |
| KOG | 64,769 | 70.85 | 75,988 | 72.41 |
| GO | 50,262 | 54.98 | 57,372 | 54.67 |
| KEGG | 48,444 | 52.99 | 55,645 | 53.02 |
| PFAM | 63,258 | 69.20 | 74,299 | 70.80 |
| Total | 91,407 | 100.00 | 104,938 | 100.00 |

**Table S7 The results from megablast search using *T. tambra* scaffolds against the NCBI database**

| **Family** | **Species** | ***T. tambra*** | |
| --- | --- | --- | --- |
|  |  | **Identity (%)** | **Coverage (%)** |
| Cyprinidae | *S. anshuiensis* | 82–97 | 11–87 |
| Cyprinidae | *S. rhinocerous* | 85–96 | 9–83 |
| Cyprinidae | *S. grahami* | 85–95 | 10–81 |
| Cyprinidae | *C. auratus* | 83–94 | 10–88 |
| Cyprinidae | *P. tetrazona* | 80–98 | 9–67 |
| Cyprinidae | *C. carpio* | 86–97 | 10–86 |

**Table S8 Statistics of *T. tambra* scaffold alignments to other organisms**

| **Organism** | **Assembly level** | **Genome length** | **Male** | | **Female** | |
| --- | --- | --- | --- | --- | --- | --- |
|  |  |  | **Coverage bases** | **Coverage (%)** | **Coverage bases** | **Coverage (%)** |
| *C. carpio* | Chromosome | 1,713,658,011 | 597,845,900 | 34.89 | 595,775,943 | 34.77 |
| *C. auratus* | Chromosome | 1,820,635,050 | 400,228,036 | 21.98 | 397,408,427 | 21.83 |
| *D. rerio* | Chromosome | 1,679,203,469 | 158,158,771 | 9.42 | 155,473,571 | 9.26 |
| *P. tetrazona* | Chromosome | 730,818,536 | 318,731,814 | 43.61 | 315,951,527 | 43.23 |
| *S. anshuiensis* | Scaffold | 1,632,718,266 | 676,300,797 | 41.42 | 671,936,468 | 41.15 |
| *S. rhinocerous* | Scaffold | 1,655,786,410 | 680,820,649 | 41.12 | 676,416,255 | 40.85 |
| *S. grahami* | Scaffold | 1,750,287,761 | 653,348,941 | 37.33 | 649,639,928 | 37.12 |
