# Supplementary figures and images for: The first de novo genome assembly and sex marker identification of Pluang Chomphu fish (Tor tambra) from Southern Thailand
Source: Comput Struct Biotechnol J. 2022 Mar 23;20:1470–80. doi: 10.1016/j.csbj.2022.03.021 (PMC8976102; doi:10.1016/j.csbj.2022.03.021)

# BUSCO Assessment Results

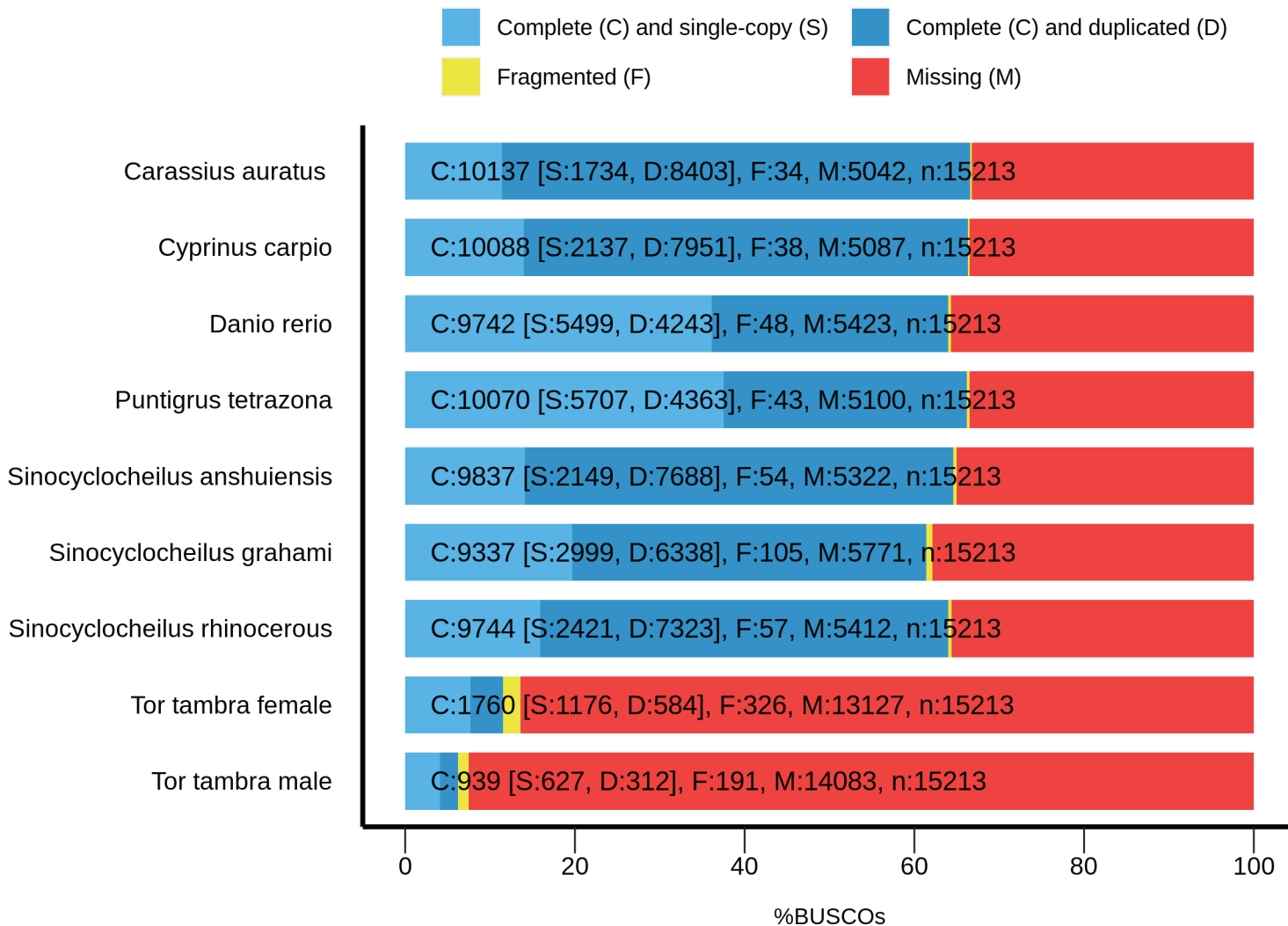

Supplement: Supplementary data 2 [file mmc2.pdf]

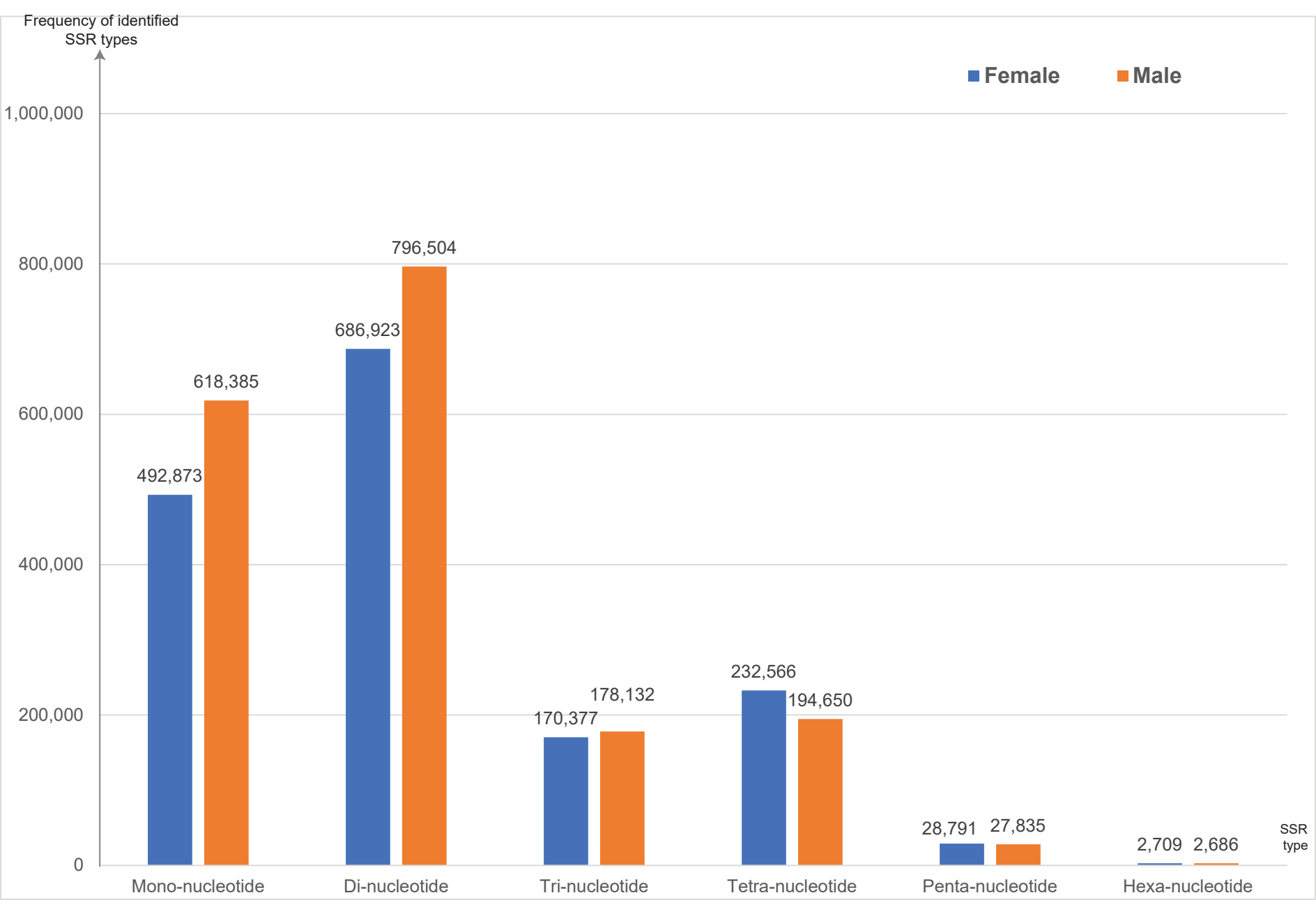

Supplement: Supplementary data 3 [file mmc3.pdf]

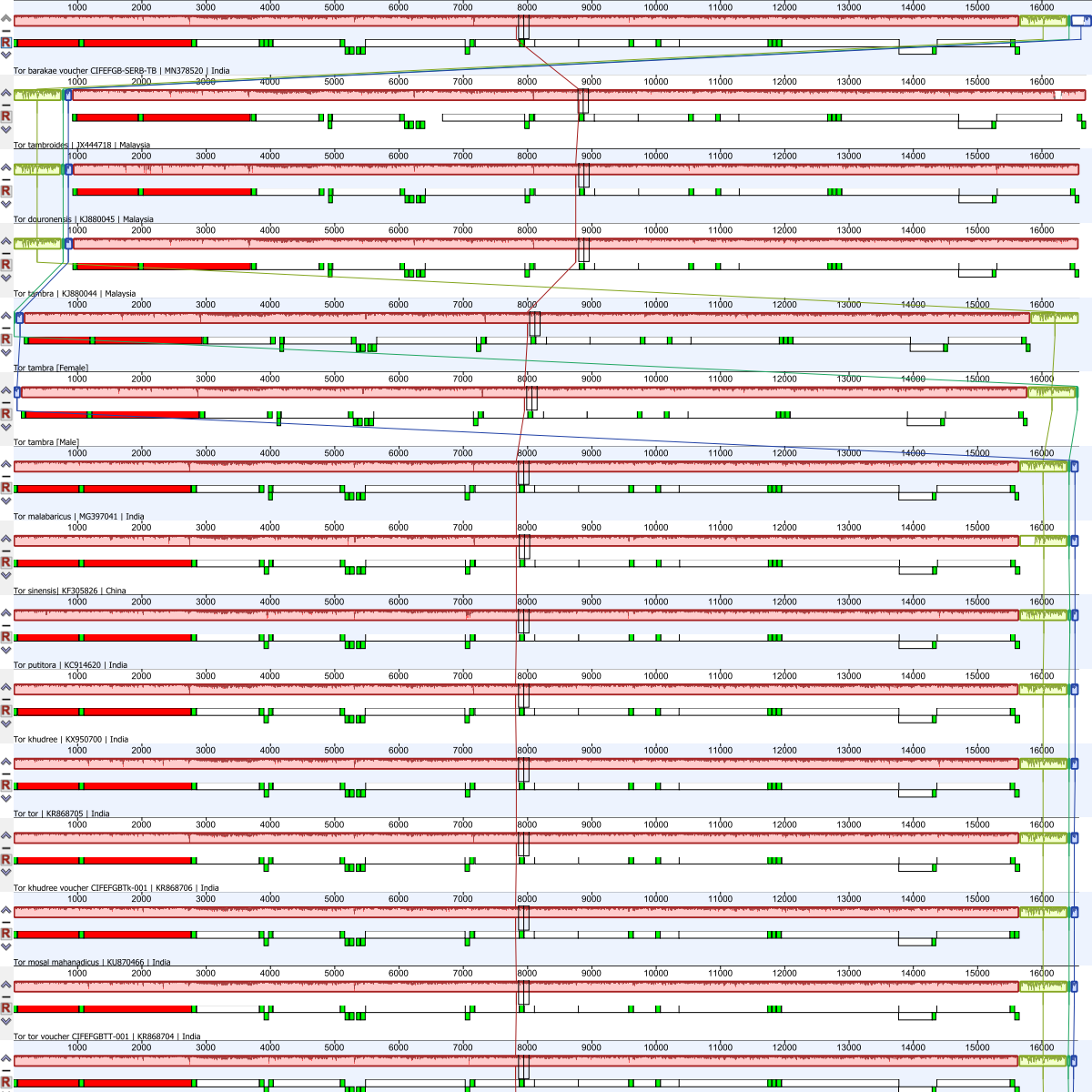

Supplement: Supplementary data 4 [file mmc4.pdf]

## Consensus Identity

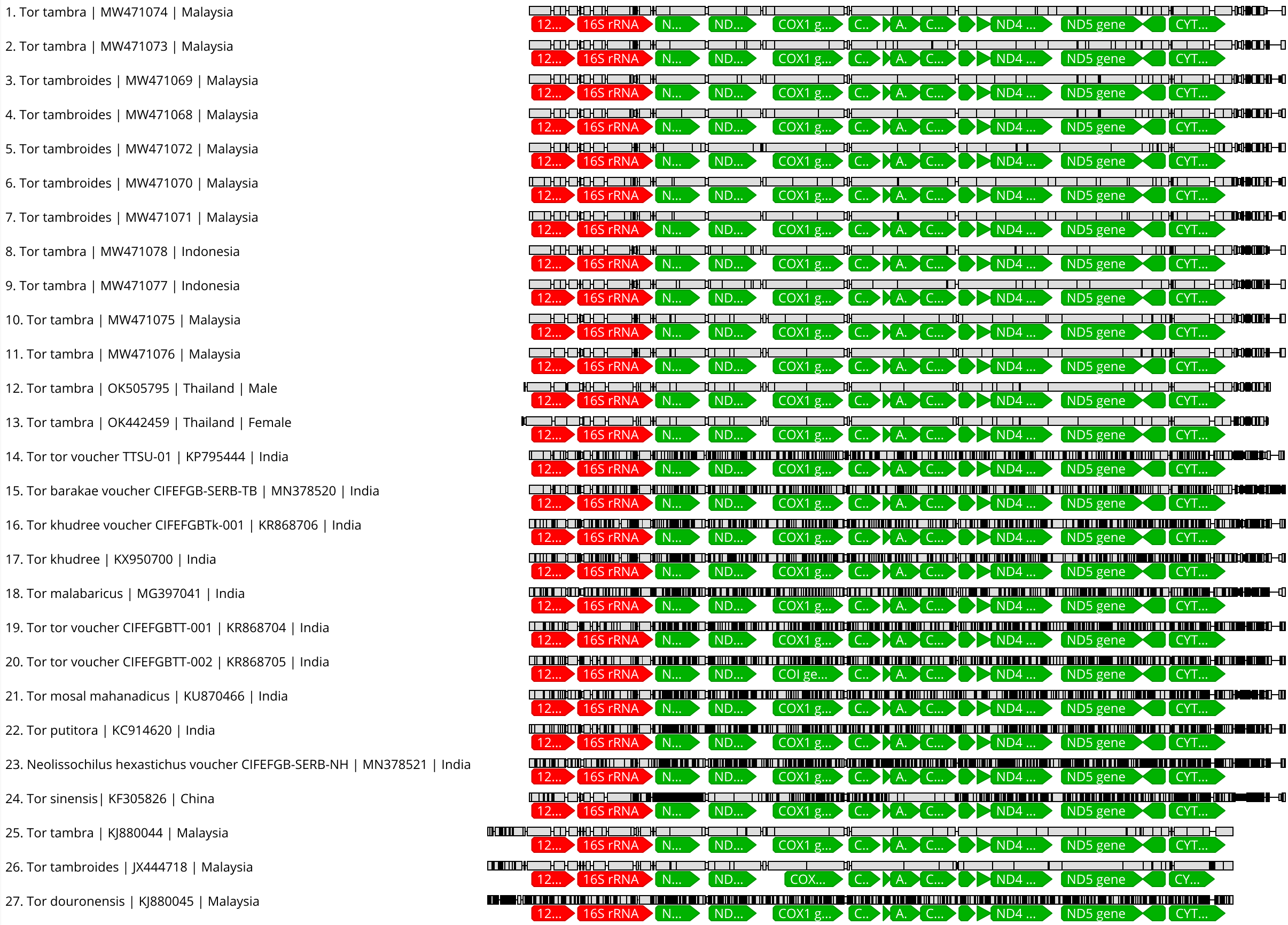

Supplement: Supplementary data 5 [file mmc5.pdf]

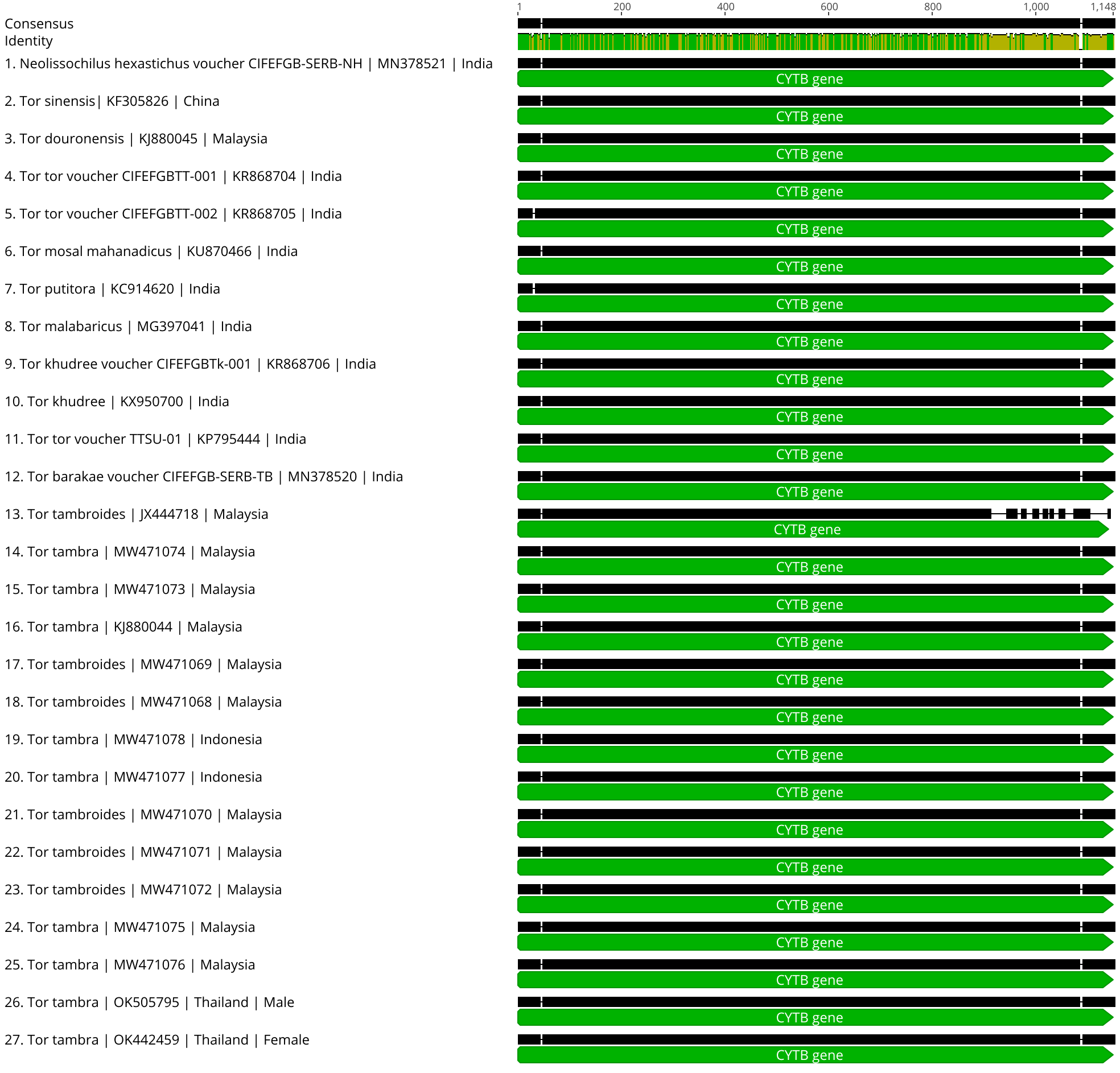

Supplement: Supplementary data 6 [file mmc6.pdf]

A

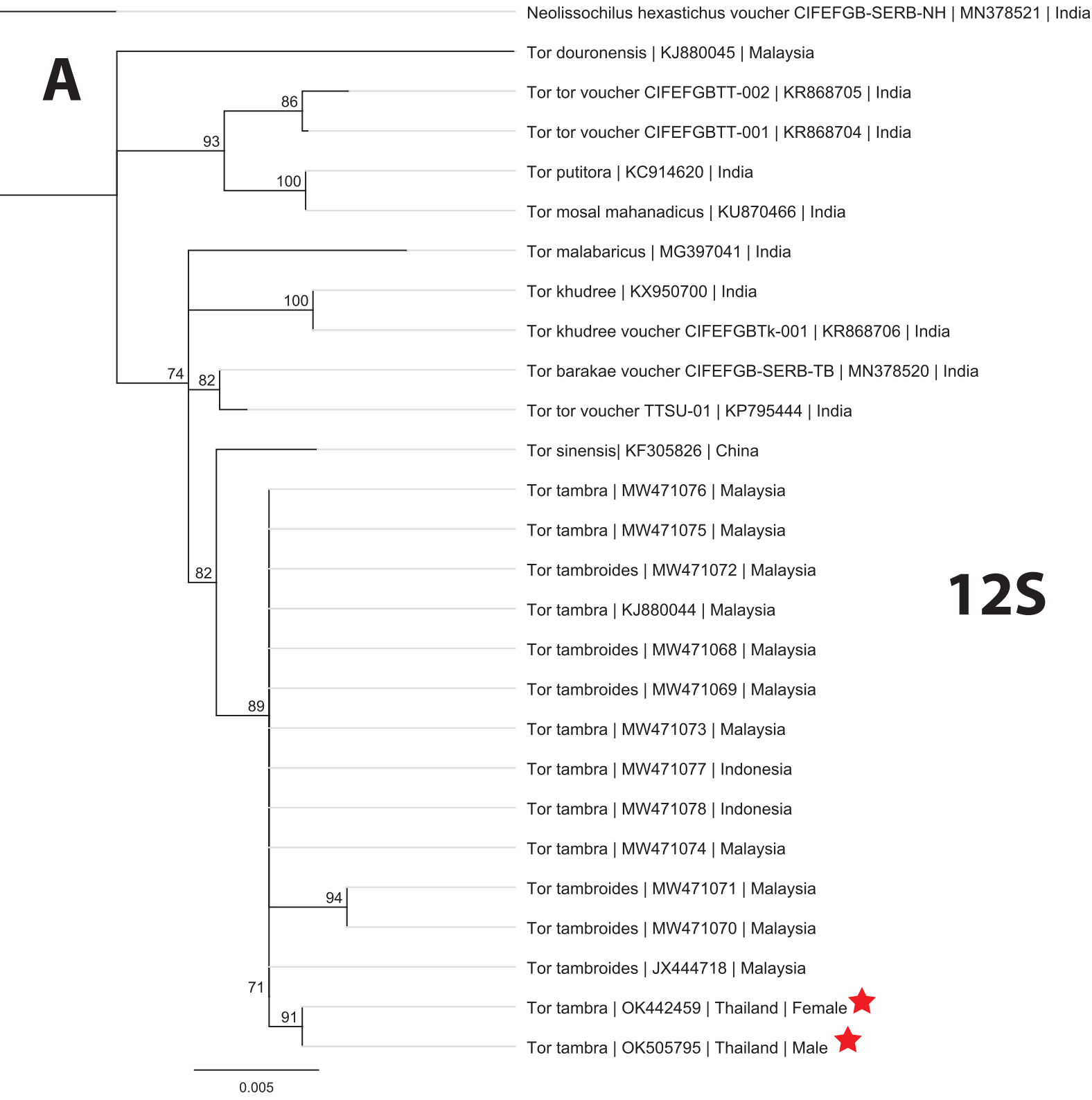

B

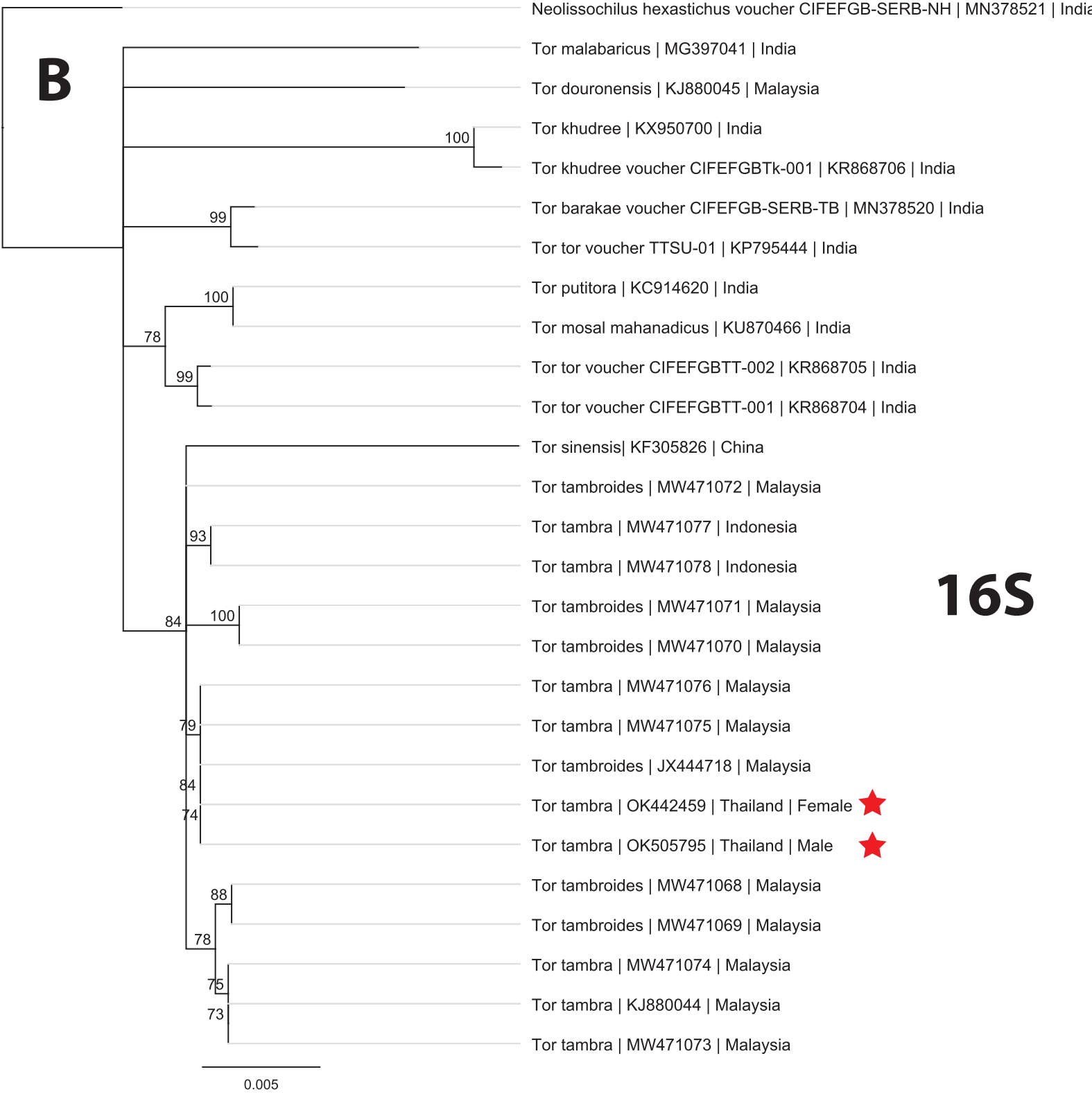

C

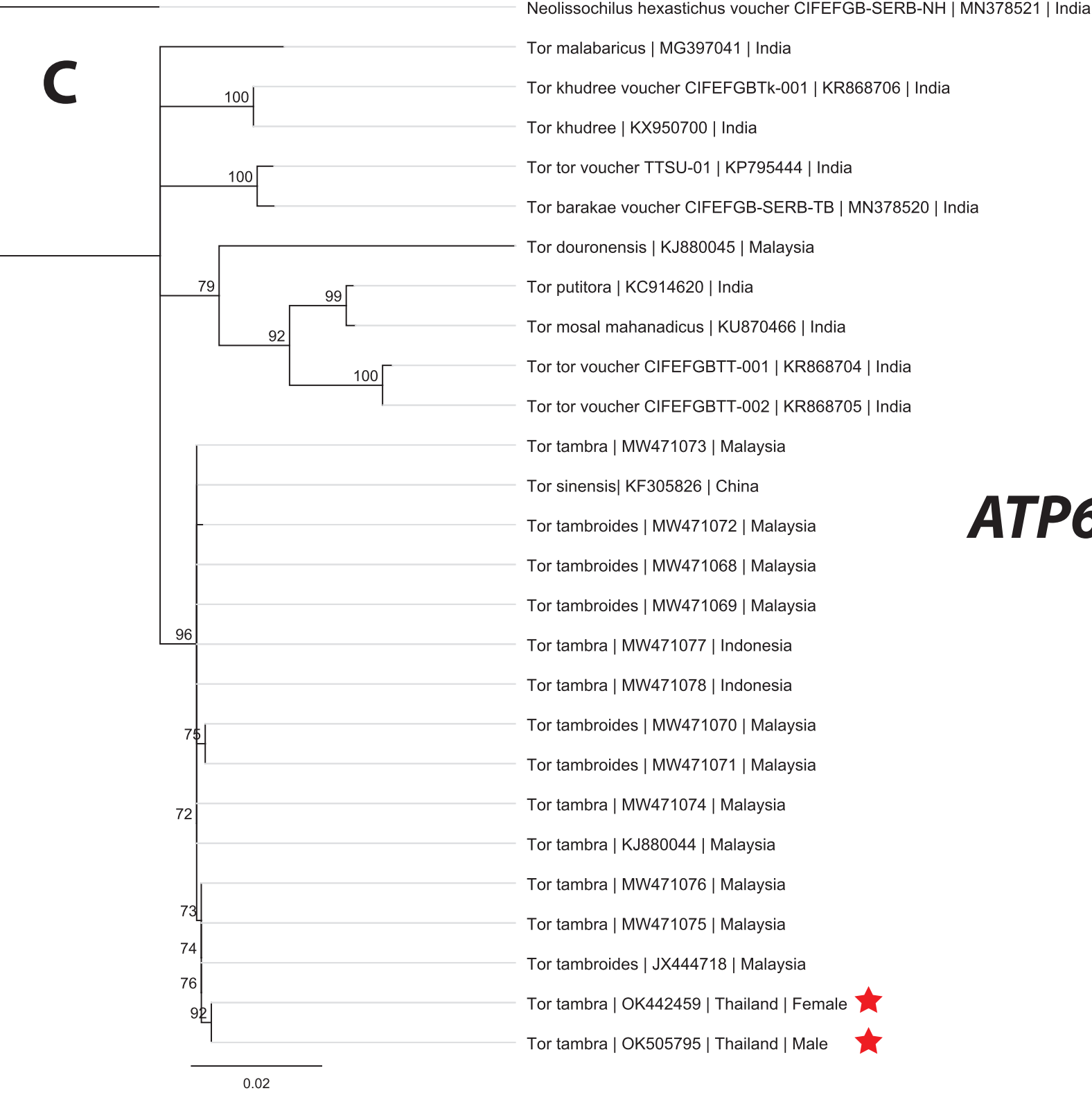

D

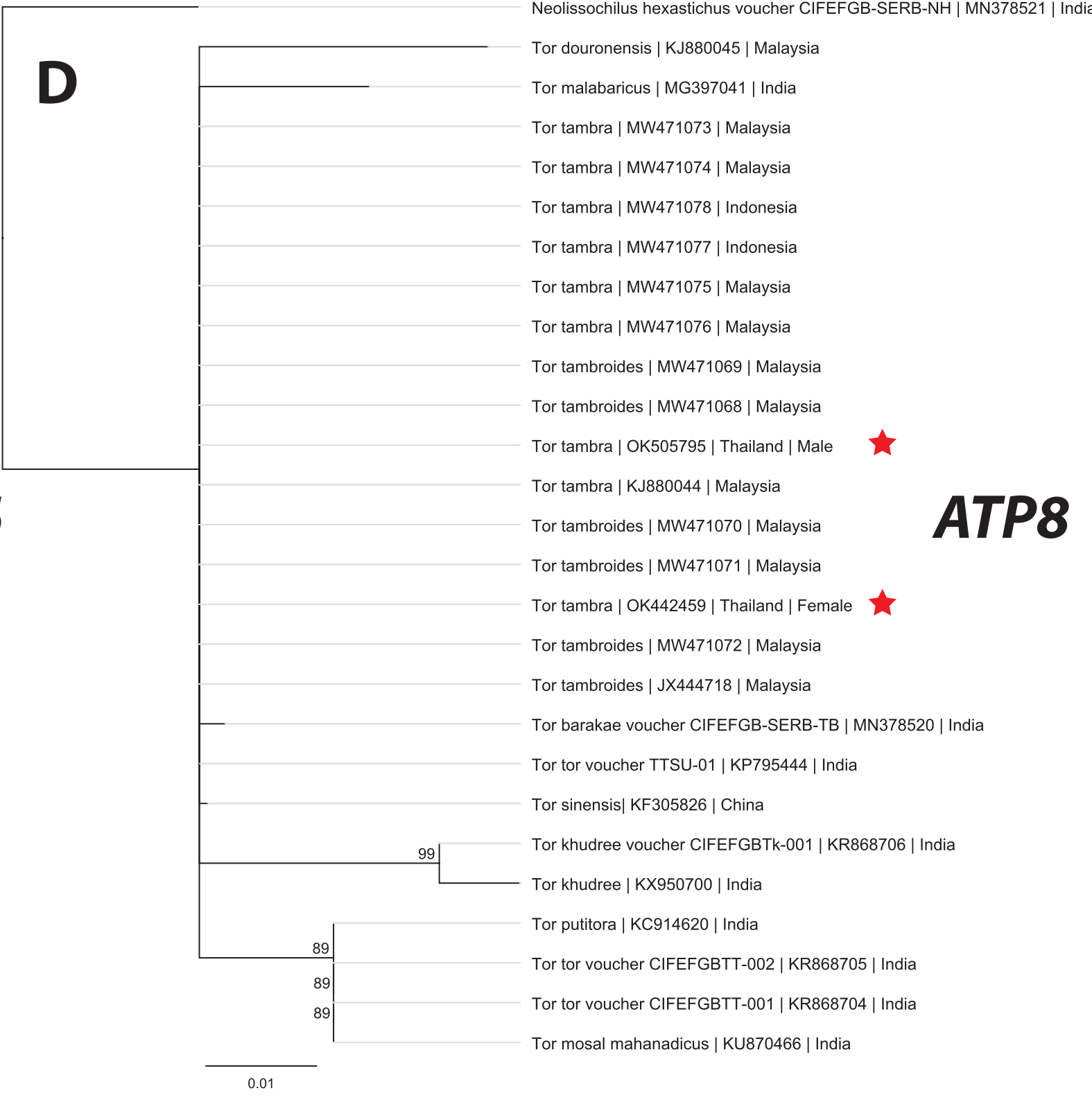

E

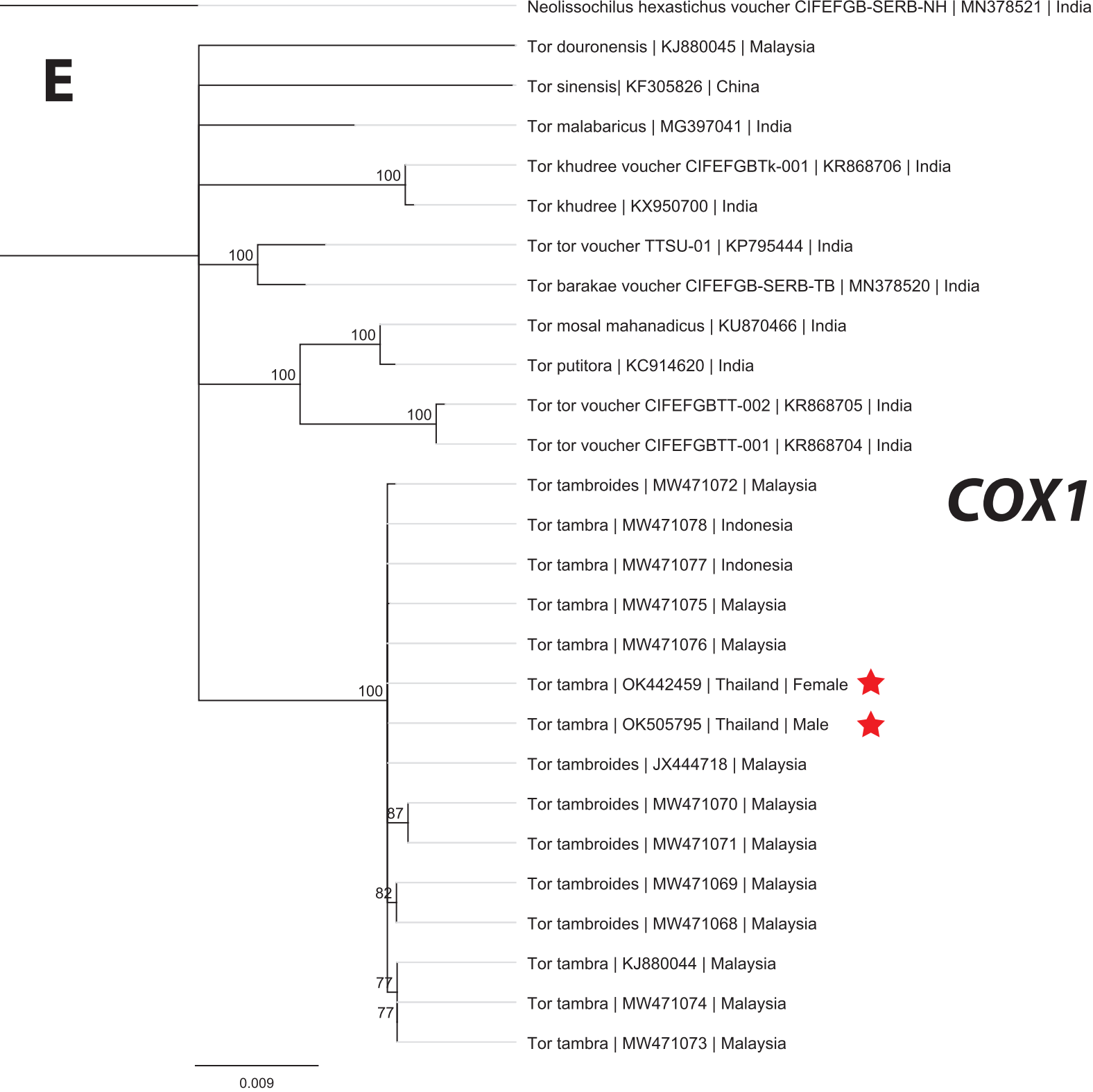

G

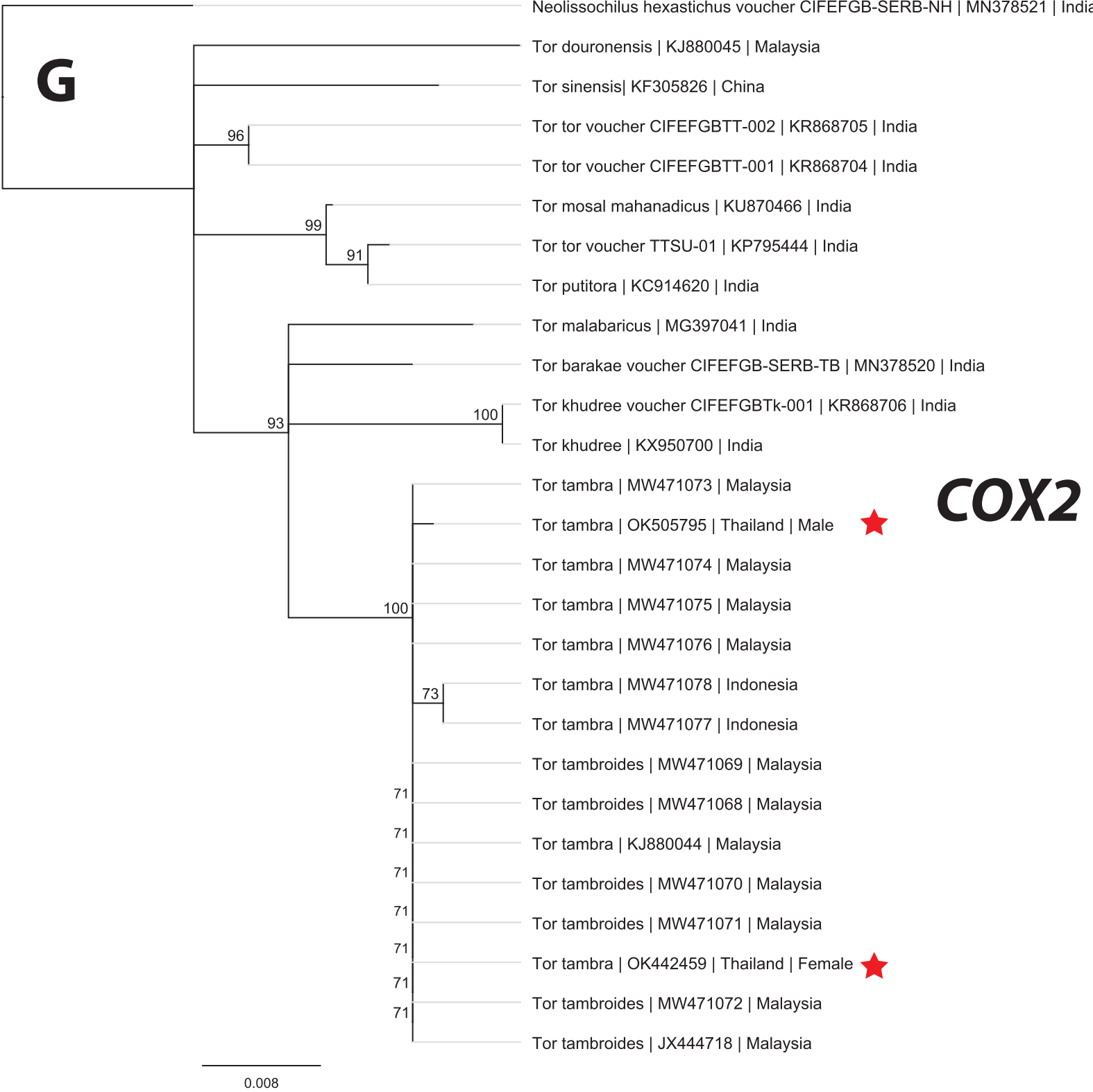

12S

16S

ATP6

ATP8

COX1

COX2



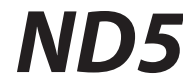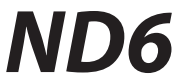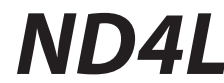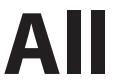

Supplement: Supplementary data 7 [file mmc7.pdf]

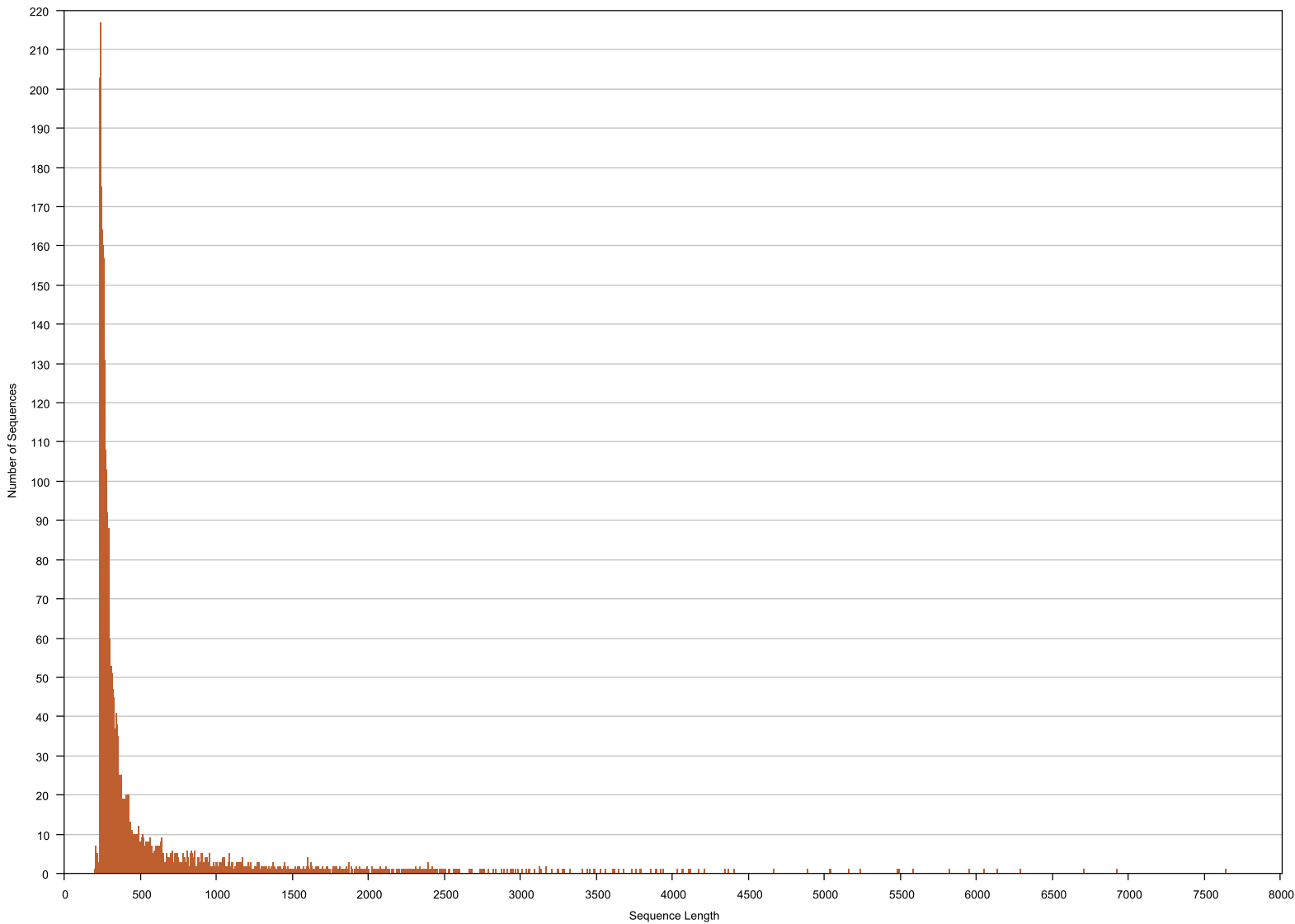

Supplement: Supplementary data 8 [file mmc8.pdf]
